# Plant-Based Seafood Analogs

**DOI:** 10.3390/molecules26061559

**Published:** 2021-03-12

**Authors:** Meital Kazir, Yoav D. Livney

**Affiliations:** Faculty of Biotechnology and Food Engineering, Technion—Israel Institute of Technology, Haifa 3200003, Israel; smmeital@campus.technion.ac.il

**Keywords:** fish, seafood, analogs, plant-based, proteins, texture, structure

## Abstract

There is a growing global need to shift from animal- towards plant-based diets. The main motivations are environmental/sustainability-, human health- and animal welfare concerns. The aim is to replace traditional animal-based food with various alternatives, predominantly plant-based analogs. The elevated consumption of fish and seafood, leads to negative impacts on the ecosystem, due to dwindling biodiversity, environmental damage and fish diseases related to large-scale marine farming, and increased intake of toxic substances, particularly heavy metals, which accumulate in fish due to water pollution. While these facts lead to increased awareness and rising dietary shifts towards vegetarian and vegan lifestyles, still the majority of seafood consumers seek traditional products. This encourages the development of plant-based analogs for fish and seafood, mimicking the texture and sensorial properties of fish-meat, seafood, or processed fish products. Mimicking the internal structure and texture of fish or seafood requires simulating their nanometric fibrous-gel structure. Common techniques of structuring plant-based proteins into such textures include hydrospinning, electrospinning, extrusion, and 3D printing. The conditions required in each technique, the physicochemical and functional properties of the proteins, along with the use of other non-protein functional ingredients are reviewed. Trends and possible future developments are discussed.

## 1. Introduction

Nowadays there is a rising global need to shift from animal- to plant-based diets, due to multiple reasons. First, the rising global population, along with high consumption of animal-based food, leads to dwindling natural resources of land and fresh water needed for sustaining the agricultural basis for this inefficient production. This leads to immense environmental pressure, diminishing biodiversity and rising environmental pollution, global warming and related adverse consequences [1,2,3]. Second, unhealthy lifestyle, which involves unbalanced nutrition and insufficient physical activity, has led to an increase in chronic diseases, metabolic syndrome, obesity and cancer [1,4,5]. In particular, high consumption of red meat has raised great concerns, due to its correlation with elevated morbidity and mortality rates [6]. Third, animal welfare is in the interests of many, with the hope of replacing traditional meat and fish production with plant-based alternatives [6]. It is noteworthy that the Food and Agriculture Organization of the United Nations (FAO) and The World Health Organization (WHO) have recommended an increased consumption of fish-meat and seafood, thanks to their high nutritional value and positive health effects [7]. Elevated marine farming of fish and seafood back-winded by these recommendations, may have negative impacts on the ecosystem due to dwindling marine biodiversity, environmental damage and fish diseases [8]. Also, discarding of unwanted catches to the sea has become a concern to the European Commission, which developed a reform in the Common Fisheries Policy, to battle this environmentally irresponsible behavior, by introducing landing obligation [9]. Furthermore, several previous works indicated that reducing fish and seafood consumption may prevent the risk of toxic substances intake, such as heavy metals, mercury from tuna fish in particular [8,10,11,12], which results from environmental water pollution. These facts, along with the consequent rising dietary shifts towards vegetarian and vegan lifestyles, encourage the development of plant-based analogs to fish and seafood, mimicking the texture and sensorial properties of fish-meat, seafood or processed fish products.

The market of plant-based meat analogs, or meat alternatives, has been steadily increasing [13,14,15,16]. Intense research and product development efforts are aimed at mimicking the structure, texture or sensorial properties of whole-muscle meat, or processed meat products, such as burgers, patties, sausage, and nuggets, including the ones of fish-meat and seafood [14,17]. Mimicking the structure, texture and sensorial properties of fish-meat is an emerging niche in the field of meat analogs. Vegans and vegetarians, who avoid animal-based seafood for humanitarian and sustainability reasons, but like their taste and nutritional benefits, would be able to enjoy highly similar alternative foods, not requiring the killing of animals. People who consume ‘Kosher’ foods will be able to enjoy the taste and texture of seafood, without violating their religious rules. The environment, and consequently future generations, will benefit from less disturbance to marine ecosystems, and better sustainability.

Mimicking the internal structure and texture of fish or seafood requires simulating their nanometric fibrous structure, resulting from the tissue-, cellular and molecular level structures, particularly from intra- and intermolecular bonds between protein chains. Some works have integrated plant protein isolates or concentrates, mainly from soy and pea, into surimi gels, through partial or full substitution of fish raw material or fish myofibrillar proteins [18,19,20]. A number of companies are already developing such products (Table 1), with some already out on the market. Most companies do not aim to imitate the structure and texture of fish or seafood, but to imitate the sensorial properties of processed fish products, in terms of appearance, texture, smell and taste.

The aim of this short review is to summarize some guiding principles for mimicking the internal structure and texture of seafood and fish meat, whole-muscle in particular, from plant-based raw materials, particularly proteins.

## 2. The Objects of Imitation: Fish, Surimi and Other Seafood Products

### 2.1. Fish Flesh

**Figure 1:**
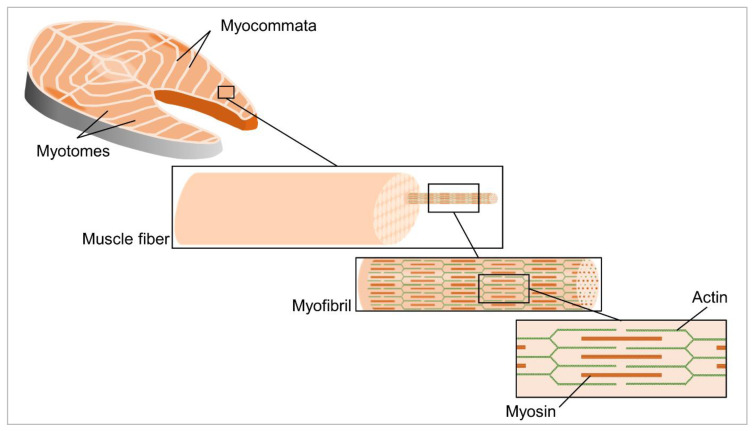
Structural organization within fish flesh.

Fish flesh main constituents are water (70–80%), proteins (15–20%) and lipids (2–5%). Carbohydrates, minerals and vitamins are also present, though in smaller concentrations, and make up about 2% of the flesh mass [21]. Fish muscle is mostly composed of myotomes, which are muscular sheets connected to one another by connective tissues, called myocommata [22,23]. Myotomes are composed of mainly myosin and actin, which together form a large number of single muscle fibers [21]. Thin actin and thick myosin filaments form myofibrils, a sub-muscle fiber with alternating anisotropic and isotropic structures, and a diameter of up to 5 µm [21]. Myofibrils form bundles with a diameter of 0.02 to 1 mm, and a length of less than 20 mm, which constitutes the muscular sheets of the myotomes [21] (Figure 1). The most abundant protein in the myocommata connective tissue is collagen, which comprises 3–10% of the proteins, and has a major role in maintaining the fillet structure and muscle cohesiveness [24].

### 2.2. Surimi

Surimi, which is often shaped into typical seafood shapes, is a food gel generated from myofibrillar protein concentrates, extracted from chopped or minced fish muscles after a washing and refining process [25]. Surimi contains ~16% protein (wet weight basis) [26], and has been widely used to produce different molded or restructured seafood products, thanks to its gelation ability [18]. During the refining process of surimi, salt (NaCl, 2–3%) is usually used to enable the extraction and unfolding of myofibrillar proteins. Upon heating, during the reconstruction process of surimi, a viscous paste of these proteins is formed, which undergoes heat-induced gelation, by the formation of intermolecular bonds and a three-dimensional protein network [27,28]. The intermolecular bonds formed during gelation can be hydrogen bonds, ionic interactions, hydrophobic interactions, or covalent- mainly disulfide- bonds [29]. Myotome-like fibers are developed, which contribute to obtaining final products with the desired textural properties, mimicking those of fish- and other seafood meat [28].

### 2.3. Processed Fish Products and Surimi-Based Products

Processed fish products can be produced from fish-meat or from surimi. Common surimi-based products among Asian population are kamaboko (a fish loaf made of cured surimi) and chikuwa (a cooked jelly-like food product made from fish surimi, salt, sugar, starch, monosodium glutamate, egg white and flavoring agents). Other common processed fish and seafood products are fish sausages, burgers, cakes and crab meat patties. Such processed fish-meat products were first developed and introduced in south-east Asian countries a few decades ago, and have since become very popular all over the world [30]. The processed fish products are characterized with resilient texture [30], obtained mostly through cold gelation, which is convenient for molding after the heat treatment. Often, additional binding agents are used to induce gelation. The most widely used binding agents are alginates, which form a polysaccharide gel entrapping the protein, or microbial transglutaminase, which crosslinks the protein itself to obtain a stronger gel [31].

## 3. Textural and Sensorial Properties of Fish

### 3.1. Texture

Texture is a humanly perceived sensory trait, related to the rheological properties of the product. It is critical for the overall quality and acceptability of a food product by consumers, particularly when mimicking fish fillet and fish-meat products [32,33]. Therefore, the manufacturing process has to lead to the desired texture profile of the product. Also, evaluation of the texture profile of a product should be reliable, robust and simple to perform [32]. Instrumental methods of monitoring texture are not only more repeatable and objective, but also less costly than a sensory quality panel [22,33], hence are useful for quality assurance during production. However, they cannot replace consumer acceptance sensory evaluation during product development.

Fish-meat is characterized by its fibrous gel structure and texture, where the techniques used to achieve this typical structure, in restructured fish products, have been previously divided into two strategies: bottom-up and top-down [6,34,35]. In the bottom-up strategy, the structural organization of a muscle-like structure is created gradually. First, protein fibers are assembled, to obtain fibers with similar dimensions as myofibrils. Then, fibers are aligned and cross-linked, to create an anisotropic gel structure [36]. In the top-down strategy, the three-dimensional structure is obtained by structuring of biopolymer blends using an external forcefield, facilitating the formation of an anisotropic structure [34]. Techniques among the first strategy are cultured meat production using tissue engineering, production of filamentous fungi, wet spinning and electrospinning, whereas among the second one are extrusion, freeze structuring and shear cell technology [34,35]. It should be noted, though, that the categories of bottom-up and top-down are not mutually exclusive, as most techniques involve both bottom-up and top-down elements. For example, extrusion, which is considered a top down method, relies on heat-induced protein aggregation and phase separation which are bottom-up self-assembly processes, while electrospinning, which is considered a bottom-up method (as nanofibers are first formed, and later used to form a 3D structure), actually uses external force fields of extrusion and electric field-induced elongation, to form the nanofibers.

Fish muscle texture is affected by several factors. First, increased myofibrillar protein and collagen content result in a higher tensile strength [37]. Second, microbiological and autolysis processes occurring postmortem, result in myofibrillar protein degradation and hence softening of the muscle [38]. In general, soft flesh is less accepted by consumers, compared to a firmer texture, and is considered less fresh [37,38]. Third, the texture of a fish fillet varies with fish type, age, geographic location, growth conditions, and anatomic location [22,37,39]. Wu et al. [39], have shown that texture profile analysis (TPA) parameters vary drastically between different parts in one sample of salmon fillet, indicating that a salmon fillets had mixed constituents and non-uniform distribution of texture. The fourth factor affecting the texture of fish-fillets and fish-meat products is the cooking method. Fish-fillets, such as salmon for example, are sometimes eaten raw or after a low-heat smoking or cooking [40]. Upon cooking, myofibrillar proteins denature, which results in changes in fish fillet texture. Cooking also changes other sensorial properties, such as taste, smell and color [41].

Currently, the standard and most widely used instrumental method to evaluate fish texture is a “Texture Analyzer”, which measures the response of the product to force exerted [22]. Examples for effects of fish source, cooking method and other factors on the texture profile of the final product are provided in Table 2.

### 3.2. Appearance

Appearance of a food is often the first attribute to attract the interest of a costumer [46]. Fish-meat analogs mimicking the structure and texture of fish-muscle should also resemble the imitated product in appearance. Seafood and fish appearance comprises several attributes, including color, shape, visual texture and uniformity [47]. Color differs greatly between seafood products. Fish fillet color ranges between red, yellow and white tones [47,48]. Therefore, addition of a colorant such as astaxanthin (Salmon pigment, which is also a potent antioxidant), or other carotenoids, may be effective in attributing the analog with the typical reddish tones [49]. Restructured fish products, such as fish burgers, should have a dark color post-cooking, stemming from lipids, blood pigments and soluble nitrogen compounds in the whole fish [46]. In terms of shape, restructured seafood products produced from minced or chopped fish can be shaped into burgers, patties, sausages, nuggets or any desired shape. Fish-muscle analogs should adapt the typical muscle appearance, including flakiness stemming from muscle fibers held together by connective tissues [50].

### 3.3. Flavor

The wide range of typical flavors of fresh fish and seafood products are uniquely distinguishable from terrestrial animal-based food. They are also characterized by Umami, the fifth basic taste, which is described as savory and brothlike [51]. Fish and seafood flavors are derived from both volatile and nonvolatile components. The concentration of both volatile and nonvolatile components in fish and seafood vary with species, age of the aquatic organism, environmental conditions, and product freshness [21,52]. The volatile compounds include mainly carbonyls and alcohols, which are formed as a result of long-chain polyunsaturated fatty acids oxidation by lipoxygenases, and organic acids. Nonvolatile compounds affecting fish and seafood flavor include nitrogenous compounds, like free amino acids, peptides and nucleotides, as well as urea and trimethylamine oxide (TMAO) [21]. Among free amino acids, glutamate, glycine, alanine, and aspartate were found to intensify the umami taste and contribute a sweet taste [21,51].

## 4. Plant-Based Proteins (PBP) Used to Mimic Muscle Structure and Texture

The main PBP sources used to produce meat analogs are legumes and pulses (soy, peas, lentils, chickpeas), pseudocereals (quinoa, buckwheat), grains (wheat (gluten), rice, sorghum), tubers (potatoes), seeds and nuts [13,53,54,55]. PBP have been used in the production process of restructured meat products, meat analogs and restructured fish products, as an additive or replacement for animal protein. PBP are used alone, or as a part of a PBP blend, to obtain a final product with texture and structure, which resemble those of the imitated animal product. For example, soy proteins and pea proteins are often mixed with wheat gluten to obtain a meat-analog, or other fibrous-structured products [56,57,58].

The use of PBP enables reducing production costs while adding nutritional value to these products, thanks to the presence of bioactive compounds [59]. Plant-based diets or diets higher in plant foods, rather than animal foods, have been recently associated with a lower risk of cardiovascular morbidity and mortality in the general population [60,61], promoting weight loss and preventing overweight and obesity [62,63]. Also, intake of micronutrients like vitamin C, vitamin E, magnesium, folate and potassium in particular, was found to be higher when consuming plant foods [16,64].

To provide a real alternative, plant-based seafood analogs should provide a similar nutritional value, mainly in terms of amino acid composition. To date, the most used PBP are soy proteins, which are considered of high quality, containing all essential amino acids, and having a high protein digestibility—corrected amino acid scores (PDCAAS) [55,65]. Combination of several PBP may elevate the nutritional value of the food product, particularly in terms of complementary amino acid composition.

The use of plant proteins not only provides the protein content in the food product, but also confers important functionalities, such as gelation, emulsification, water holding and oil binding, which are essential for meat-analog structure formation [14,55]. Plant proteins are usually globular in their native state. Therefore, to form the typical fibrous structure and texture of fish-meat, PBP must be denatured and unfolded, aligned and cross-linked [66]. Cross-linking of unfolded proteins may be obtained through hydrophobic forces, van der Waals, hydrogen bonding, disulfide bonds, electrostatic forces, enzymatic reaction, etc. [67]. Common structuring techniques of plant-based proteins into fibrous structure include hydrospinning (or wet spinning), electrospinning, extrusion and 3D printing. To date, forming fibrous-structured products from plant proteins, using these techniques, is predominantly used to produce meat analogs.

In terms of waste management, PBP are often by-products of processing raw plant materials. For example, elevated protein content of up to 23% was found in the seeds of various fruits [68]. Side streams of oil extraction from oil seeds yield protein-rich flours, containing over 50% protein [69,70]. Also, soy milk production by-product (Okara) contains up to 37.5% protein [71]. Hence, with careful purification, by-product proteins from plant processing could be utilized as raw materials in the production of fish-meat analogs, which would help reducing waste, while valorizing the raw material.

## 5. Non-Protein Texture-Functional Ingredients in Fish Analogs

Non-protein ingredients should also be used when producing fish and seafood analogs, to successfully imitate their nutritional and chemical composition, and their sensorial properties. In addition, many of these components have a significant impact on the texturization process, and other functional properties, influencing the final structure of the product.

### 5.1. Ions

Salts exert both ion-specific and non-specific effects on protein solubility, denaturation and gelation, and hence on structure and texture during thermal or shear processes, often applied to generate protein fibers [27]. With the addition of salts, and at high ionic strength, protein solubility decreases due to salting out effects, whereas low ionic strength generally enhances protein solubility. Depending on the charge distribution within the protein, long protein fibrils are typically formed at low ionic strength [72]. Ion-specific effects may also be harnessed. For example, depending on the protein and salt used, addition of certain ions may promote gelation, generating a more rigid structure. For example, addition of calcium and magnesium to soy protein, was found to promote the formation of a firmer gel [73]. Ammonium sulfate was shown to induce stronger gel formation from fish gelatin, as microstructures of hydrogels were more compact and water binding capacity increased [74]. A similar effect was observed in the case of adding calcium ions during the production process of surimi-based gels [75].

### 5.2. Lipids

Lipid content of fish ranges from 2% to 20% and varies according to species, anatomical position, season, and diet of the fish [21]. Fish lipids are mainly composed of unsaturated fatty acids. Polyunsaturated fatty acids (PUFAs) in fish are of an ω-3 type, like DHA and EPA, which are especially essential nutritionally [21]. In the production process of surimi, a stable emulsion is produced, combining fish muscle proteins and added lipids [28]. Lipid droplets may act as active or passive fillers in the gel matrix, i.e., participating, or not, in its network, thereby more or less affecting the texture respectively [76]. Reportedly, the addition of fat to surimi-based gels significantly increased breaking force, gel water-holding capacity, storage modulus (G′) and loss modulus (G″) [76,77]. The addition of fat also increased whiteness of surimi-based gels, by enhancing light scattering, thereby improving its likeness by trained and non-trained panelists [77,78].

### 5.3. Dietary Fibers

Using dietary fibers as additives in the process of fish analogs production is beneficial not only in terms of nutrition and health, but also in improving textural properties [79].

Dietary fibers added to restructured seafood products are mostly soluble, and are chosen according to their functional properties, such as water holding, emulsifying, thickening, or gel-forming [28]. Numerous studies have investigated the influence of incorporating dietary fibers into surimi-based products [79,80,81,82]. Dietary fibers can act as fillers, binders or extenders [80]. For example, inulin was found to act as a filler, filling the network gaps in a surimi-based gel [80]. Similarly, nanosized insoluble okara dietary fibers were incorporated into surimi and filled the gel matrix [82]. Overall, addition of dietary fibers improved gel strength, texture and water-holding capacity of surimi-based gels [79,80,81,82].

## 6. Structuring Techniques

### 6.1. Extrusion

**Figure 2:**
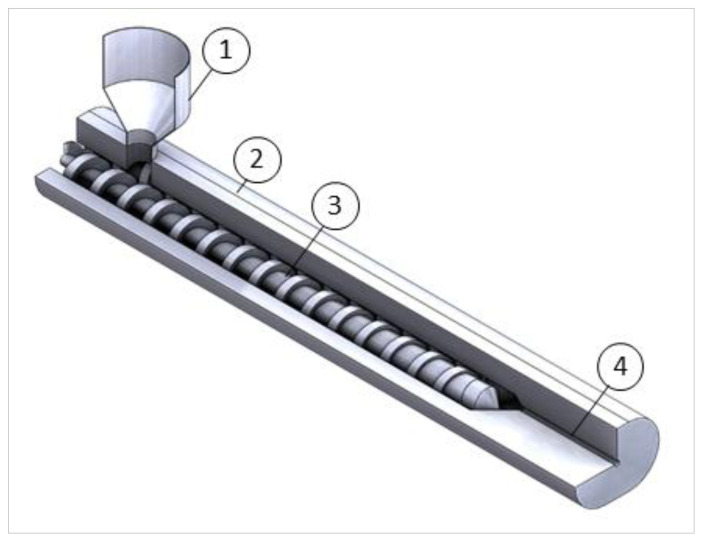
Co-rotating twin-screw extruder scheme. (1) Feed (2) Barrel (3) Left screw (right screw mostly hidden) (4) Cooling die.

Extrusion is the most common technique to transform proteins, plant-based proteins in particular, into fibrillar structure, resembling the one of whole-muscle meat or restructured meat products [14,34]. During the extrusion process, a food mixture containing proteins is fed into a barrel containing a rotating screw, while heat, high pressure and shear are applied. Then, the sheared molten mixture is pushed (extruded) through a cooling die (Figure 2) [55].

Protein extrusion is classified into low-moisture extrusion (<30% moisture content) and high-moisture extrusion (>50% moisture content). The first is mostly intended to produce texturized vegetable protein (TVP). TVP could be rehydrated, after extrusion, and used for the production of meat analog products as chunks, nuggets and shapeless crumbles, resembling ground or minced meat [14]. High-moisture extrusion is used to produce whole-muscle meat texture, characterized by fibrous, anisotropic structure. The high moisture content, laminar flow and long cooling die, enable the formation of such anisotropic structure [14,83]. Several factors affect the final product properties: extrusion temperature profile, screw speed, extrusion pressure, energy input and die geometry [14,84]. Also, the presence of polysaccharides, such as starch, and functional groups in the protein, such as disulfide bonds, would affect the structure and texture of the extrudate [84,85,86].

Extrusion of plant-based proteins into fibers with an anisotropic structure, for the production of meat analogs was described in numerus papers. Recent papers examined new plant-protein sources, such as peanuts, in a blend with more traditional plant-protein sources, such as soy, or with polysaccharides, to form fibrous structures [87,88,89]. In general, it was concluded that an anisotropic structure could be obtained with high extrusion temperature [84,90]; However, when combining high temperature with high screw speed, a porous extrudate may be obtained, due to boiling of water upon pressure release at the die exit [84]. To obtain a fibrous structure under high temperature and screw speed, moisture should be kept low [88].

### 6.2. Electrospinning

**Figure 3:**
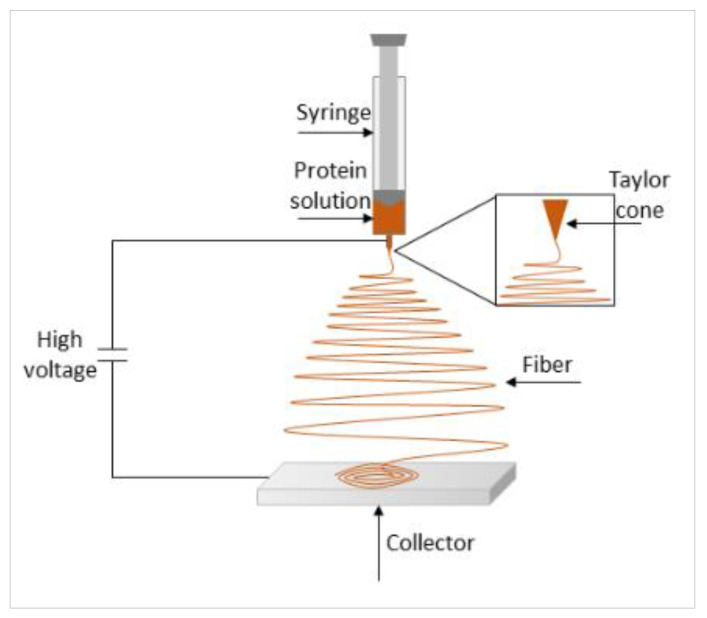
Principle and typical setup of electrospinning.

Electrospinning is the structuring of polymer solutions into nanofibers using high voltage. The protein solution is pushed through a nozzle, and is electrically accelerated by the electric potential gradient relative to the ground electrode. As a consequence, the fine jet emerging from the nozzle, in a Tylor cone form, extends into a thin fiber while the solvent evaporates and is finally collected on the collector, connected to the ground electrode (Figure 3) [34,36,55]. In order for the electrospinning to occur, spun proteins must be in unfolded conformation, or intrinsically unstructured, rather than globular. They should also be highly soluble and sufficiently concentrated. In a globular conformation, proteins may not sufficiently overlap, leading to fewer interactions with each other, and insufficient entanglements for a fiber to form [34,36]. To overcome this problem, higher concentrations of protein may be used in the process, though not exceeding the maximal solubility. Plant proteins are usually globular in their native state, therefore, they should be unfolded (usually using heating) prior to electrospinning, while avoiding the formation of insoluble aggregates [34]. Another solution is to combine the proteins with a ‘spinnable’ polymer. Electrical conductivity and viscosity greatly influence both the electrospinnability of the protein solution and the morphology of the formed fiber [91]. Increased viscosity stabilizes the jet due to entanglement of polymer chains, resulting in the formation of a continuous fiber, rather than a spray of droplets [91]. Increasing protein concentration increases electrical conductivity and viscosity. However, the viscosity should not be too high, to ensure flowability of the protein solution [36]. In addition, Moreira et al. (2018) [92] have found that an acidic spirulina protein solution enabled the formation of smooth, long, fine fibers (for potential application in the food packaging field), with minimal occurrence of droplets and agglomerates, as opposed to using an alkaline solution. Plant-proteins have been widely used to form fibers through electrospinning. In particular, zein electrospun fibers were intended to mimic the desired texture of meat and produce meat analogs [36,93].

### 6.3. Wet Spinning

In this process, a protein solution is extruded into a coagulation bath, containing a solvent, which down-shifts protein solubility, or promotes cross-links and fiber formation. The solvent can be such that causes protein precipitation, and together with the high shear forces at the nozzle, they cause alignment of the proteins, to form stretched filaments [34]. To promote cross-links, the solvent should contain binding agents, such as Ca^+2^, or provide an environment (e.g., pH) that would promote the formation of inter- and intramolecular bonds between protein chains. The fibrous material formed is then collected, and washed from the solvent [55]. Properties of the pre-spun solution play an important role in the process. Mu et al. (2019) have found that at the same concentration of protein in solution, higher spinning temperature increased the mobility of protein molecules, thereby weakening the molecular interactions and entanglement [94]. In addition, Liu et al. (2017) have found that higher concentrations of protein, and increased temperature, facilitated spinnability of a protein solution, and resulted in stronger fibers [95].

### 6.4. 3D Printing

Three-dimensional food printing is developing fast over the last decade, and many different 3D printing techniques are available. The most common one is based on syringe injection. In this process, a protein solution, with a very high viscosity, is extruded through a moving fine syringe nozzle, layer by layer, to form a 3D product, e.g., a muscle-like structure [14]. The print is based on a pre-designed digital model [25,96]. The printed protein solution, used as ‘ink’, should be homogenous and have appropriate printability [97]. The printability refers to physical and chemical properties, ensuring its flowability out of the nozzle, and capability of maintaining and quickly rigidifying the 3D structure post-deposition [96,98]. Fish-meat products are eaten raw, or after cooking; Therefore, the printed 3D model should be durable and resistant to thermal cooking processes post-deposition [98].

Optimized printing parameters are chosen according to the desired final structure and physical properties of the printed model. To generate a fine fibrous structure with small diameter fibers, resembling the ones of fish-meat, smaller diameter nozzle is preferred. High printing speed and low nozzle height impair the deposition of the printed solution, hence reduce printing precision, and result in a product with poor mechanical strength [99]. However, Wang et al. (2018) showed that at very low moving speed, the flow instabilities of the printed slurry resulted in the formation of a less accurate print. In addition, at very high nozzle height, the printed fibers were much thicker than intended [25].

The chemical composition of the printed solution affects not only its printability, but also the structure, physical and mechanical properties of the final product [25]. For example, the presence of carbohydrates may increase solution viscosity, to such an extent that may impair its flowability during printing, but the molecular interactions of the carbohydrates with the proteins may facilitate the formation of a fibrous structure. 3D printing of soy protein mixed with alginate and gelatin improved the hardness and chewiness of the printed product [100]. Higher concentration of salt, particularly NaCl, was found to increase the uniformity of the printed slurry, resulting in less broken deposited lines [25]. NaCl was also found to decrease the viscosity of a soy protein isolate mixture with xanthan gum, leading to improved printability [101]. Plant-proteins, e.g., soybean proteins, can also be applied in printing to generate a porous scaffold, mimicking extracellular matrix. The scaffold is then used to support cell attachment and proliferation, creating a 3D engineered cultured muscle tissue [102].

## 7. Conclusions

Plant-based seafood analogs, mimicking the structure, texture and sensorial properties of seafood, fish-meat and other processed fish-meat products, comprise an innovative new and growing niche in the world of meat analogs. Strategies and techniques applied to produce plant-based meat analogs can inspire the production of seafood analogs, with necessary modifications and optimizations. These stem from differences between fish-meat and mammalian meat in terms of chemical and nutritional composition, structure, texture and sensorial properties. The chemical composition of the final product should resemble the one of seafood or fish-meat, to provide the consumer with a similar, or even better nutritional composition. In addition, the chemical composition should allow, or facilitate, the texturization process, and confer the desired structural and sensorial properties to the final product, for a satisfying consumption experience. Development of such products will contribute to a more sustainable food production, with benefits to the environment, public health and animal welfare.

## Figures and Tables

**Table 1:** Plant-based fish and seafood alternatives on the market, or under development.

Product Type	Main Ingredients	Company
Tuna chunks, fish burgers, fish cakes and crab cakes	Six-legume blend (including peas, chickpeas, lentils, soy, fava beans and navy beans)	Good Catch
Fish filet and crab cakes	Soy, wheat, potato	Gardein
Caviar	Seaweeds	Plant-Based Foods
Fish fingers, tuna pate, fish cakes, smoked salmon	soy, potato, konjac, wheat	VBites
Ahimi^®^—raw tuna, Unami™—raw eel	Tomatoes/eggplants	Ocean Hugger Foods
Shrimp	Seaweeds	New Wave Shrimp

**Table 2:** Texture profile analysis (TPA) of various fish products.

Product	Cooking Conditions	Hardness (N)	Chewiness (N)	Cohesiveness ^2^	Springiness ^2^	Resilience ^2^	Reference
Pacu fillet	Grilled for 6 min, internal temperature of 60 °C	4.91 ± 0.64	1.65 ± 0.25	0.46 ± 0.05	NA	0.17 ± 0.02	[42]
Catfish fillet ^1^	Baked at 149 °C, internal temperature of 74 °C	2.16 ± 0.34	0.77 ± 0.18	0.47 ± 0.03	73.84 ± 2.65	23.5 ± 1.79	[33]
Salmon fillet	Boiled in water for 5 min	7.43	2.36	0.29	1.00	NA	[43]
Sea Bass fillet	Smoked and dried for 3 h at 35 °C	44 ± 3	21.2 ± 1.8	0.55 ± 0.01	0.59 ± 0.01	NA	[44]
Surimi-based fish sausages ^1^	Steamed at 90 °C for 30 min, internal temperature of 75 °C	57.31 ± 0.06	5.59 ± 0.07	0.31 ± 0.00	0.32 ± 0.01	NA	[45]

^1^ Data was converted from g force to N. ^2^ Dimensionless parameters. NA = Not Available.
